# Improved clinical and laboratory skills after team-based, malaria case management training of health care professionals in Uganda

**DOI:** 10.1186/1475-2875-11-44

**Published:** 2012-02-13

**Authors:** Allen Namagembe, Umaru Ssekabira, Marcia R Weaver, Nancy Blum, Sarah Burnett, Grant Dorsey, Lydia Mpanga Sebuyira, Alex Ojaku, Gisela Schneider, Kelly Willis, Adoke Yeka

**Affiliations:** 1 Infectious Diseases Institute, Makerere College of Health Sciences, Kampala, Uganda; 2 International Training and Education Center for Health, Department of Global Health, University of Washington, Seattle, WA, USA; 3 Accordia Global Health Foundation, 1101 14th Street, NW, Suite 801, Washington, DC, USA; 4 Department of Medicine, University of California San Francisco, San Francisco, CA, USA; 5 DIFAEM, Paul Lechler Strasse 24, D 72076 Tubingen, Germany; 6 Uganda Malaria Surveillance Program, c/o Infectious Disease Research Collaboration, PO Box 7475, Kampala, Uganda

## Abstract

**Background:**

Deployment of highly effective artemisinin-based combination therapy for treating uncomplicated malaria calls for better targeting of malaria treatment to improve case management and minimize drug pressure for selecting resistant parasites. The Integrated Management of Malaria curriculum was developed to train multi-disciplinary teams of clinical, laboratory and health information assistants.

**Methods:**

Evaluation of training was conducted in nine health facilities that were Uganda Malaria Surveillance Programme (UMSP) sites. From December 2006 to June 2007, 194 health professionals attended a six-day course. One-hundred and one of 118 (86%) clinicians were observed during patient encounters by expert clinicians at baseline and during three follow-up visits approximately six weeks, 12 weeks and one year after the course. Experts used a standardized tool for children less than five years of age and similar tool for patients five or more years of age. Seventeen of 30 laboratory professionals (57%) were assessed for preparation of malaria blood smears and ability to interpret smear results of 30 quality control slides.

**Results:**

Percentage of patients at baseline and first follow-up, respectively, with proper history-taking was 21% and 43%, thorough physical examination 18% and 56%, correct diagnosis 51% and 98%, treatment in compliance with national policy 42% and 86%, and appropriate patient education 17% and 83%. In estimates that adjusted for individual effects and a matched sample, relative risks were 1.86 (95% CI: 1.20,2.88) for history-taking, 2.66 (95%CI: 1.60,4.41) for physical examination, 1.77 (95%CI: 1.41,2.23) for diagnosis, 1.96 (95%CI: 1.46,2.63) for treatment, and 4.47 (95%CI: 2.68,7.46) for patient education. Results were similar for subsequent follow-up and in sub-samples stratified by patient age. Quality of malaria blood smear preparation improved from 21.6% at baseline to 67.3% at first follow-up (*p *< 0.008); sensitivity of interpretation of quality control slides increased from 48.6% to 70.6% (*p *< 0.199) and specificity increased from 72.1% to 77.2% (*p *< 0.736). Results were similar for subsequent follow-up, with the exception of a significant increase in specificity (94.2%, *p *< 0.036) at one year.

**Conclusion:**

A multi-disciplinary team training resulted in statistically significant improvements in clinical and laboratory skills. As a joint programme, the effects cannot be distinguished from UMSP activities, but lend support to long-term, on-going capacity-building and surveillance interventions.

## Background

Many malaria endemic countries have deployed artemisinin-based combination therapy (ACT) as first-line treatment for uncomplicated *Plasmodium falciparum *malaria [1]. To improve case management and minimize drug pressure for selecting resistant parasites, ACT should be targeted to patients who are parasite positive, and another diagnosis and treatment should be sought for patients who are not. In 2010, the World Health Organization (WHO) recommended a prompt parasitological confirmation of diagnosis "in all patients suspected of having malaria before treatment is started [2]."

This recommendation has drawn attention to the poor quality of care for patients with fever in general and malaria in specific, and to a search for interventions to improve the quality of care. In a review of case management of fever among children in Africa, Zurovac and Rowe [3] reported that outpatient clinics with a recent quality improvement intervention had higher median percentages of children who were treated correctly than clinics without an intervention. In a more detailed comparison of five studies with similar research design, in-service training improved the quality of treatment for children with uncomplicated malaria in one out of five studies, guidelines in one out of two studies, wall charts in two out of four studies, and supervision visits in two out of two studies. These studies pre-dated treatment with artemether + lumefantrine (AL) or Coartem^®^, with the exception of Zurovac et al., which showed no effect of these quality improvement interventions in Zambia [4]. An update from Zambia reported increases in the percentage of children treated correctly after expanding training, wall charts, treatment guidelines and capacity for diagnostic tests [5]. A more recent study in Tanzania on the effect of introducing malaria rapid diagnostic tests and a brief training course to guide fever case management found no improvement in clinician prescribing practices [6].

Few studies have reported the effect of training programmes to improve the quality of laboratory diagnosis of malaria. Recently, Kiggundu et al. reported that a three-day laboratory training in Uganda significantly improved the sensitivity and specificity of thick blood smears, and increased the percentage of well-prepared blood smears [7]. Ngasala et al. reported the sensitivity of blood smear microscopy was 74.5% after a training programme in Tanzania, but did not report baseline data [8]. Bates et al. reported that the percentage of laboratories with accurate malaria tests increased from 84% to 91% after a training and quality assurance programme in Ghana, but did not report a statistical test [9].

The National Malaria Control Program (NMCP) of Uganda recommended AL as the first-line treatment for uncomplicated malaria in 2005 [10] due to development of resistance to chloroquine and sulphadoxine-pyrimethamine (Fansidar^®^), and initiated wide-scale distribution of AL in 2006. Accordia Global Health Foundation forged a partnership with the Infectious Diseases Institute (IDI) and the Uganda Malaria Surveillance Program (UMSP), to design and prospectively evaluate the Joint Uganda Malaria Training Program (JUMP). The evaluation used three complementary sources of data: 1) on-site observation of clinical care and laboratory testing, 2) UMSP surveillance data on four clinic-level performance indicators, and 3) quality assurance data on the laboratories. Ssekebira et al. reported the results on surveillance and quality assurance data [11]. Surveillance data for four months preceding the six-day course were compared to four months immediately after it. For both children less than five years of age and patients five years or more of age, the training programme was associated with a significant increase in the percentage of malaria suspects referred for blood smear, and a significant decrease in the percentage of patients with a negative smear prescribed anti-malarial drugs. Quality assurance data on the laboratories showed that the sensitivity and specificity of field microscopy did not improve significantly with training. In an update of the surveillance data, Figure three of Sserwanga et al. showed that the results for malaria suspects referred for blood smear persisted for more than one year after the course at three of the four sites [12] with data comparable to those reported in Ssekabira et al., and decreased slightly at the fourth site.

This article reports the results on on-site observation of clinical care and laboratory testing and makes two contributions to the literature. First, it tests the effect of the team-based training on clinical and laboratory skills relative to baseline at three time points after the course: approximately six weeks, 12 weeks and one year or more. Second, it examines the role of individual effects in a pre/post evaluation design. A key question in the evaluation of training programmes is whether or not unobserved differences across clinicians or "individual effects" bias the results. There may be differences in practice style across clinicians, which researchers can not control with variables such as profession, years of experience and in-service training. Alternatively individual practice style may be less important in a setting where the range of treatment options is narrower. Several authors have controlled for individual effects in cross-section studies of on-site observation of clinical care [13-17]. This article is the first to report results that correct for individual effects in an evaluation of a training programme with a longitudinal design.

## Methods

### Evaluation sites

Training was implemented at nine malaria surveillance sentinel sites in Uganda. UMSP established the sites in collaboration with the NMCP to monitor trends in malaria morbidity and indicators of malaria case management. A UMSP team visited each site every one or two months to supply forms for surveillance data and laboratory consumables, as well as feedback. The sites represented the diversity of geography and malaria transmission intensity in Uganda [18,19], which in turn represented the diversity of transmission intensity in Africa [20] as measured by the Entomological Inoculation Rate (EIR). As shown Table S1 of the Additional file 1 the annual EIRs of the nine sites ranged from less than one infective bite per year at two highland sites (Kabale and Kamwesi) to more than one infective bite per day at three sites where transmission intensity was holoendemic (Omugu, Nagongera and Aduku). Indoor residential spraying was conducted in households served by Kabale and Kamwesi in 2006 and 2007, Kihihi in 2007, and Aduku in 2008 and 2010 [21].

Eight of nine sites were government health centres IVs with a catchment population of 100,000 people. They were equipped with an outpatient clinic and laboratory, and according to Health Sector Strategic Plan norms should be staffed with clinicians, laboratory professionals, and health information assistant (sometimes referred to as data clerks) [22]. The ninth site was a government regional referral hospital with a catchment population of approximately two million people and a larger staff. Outpatient care is free of charge at all sites. In 2006, staff at the sites attended a one-day training on the new NMCP recommendations for AL organized by the Ministry of Health.

### Intervention

**Figure 1:**
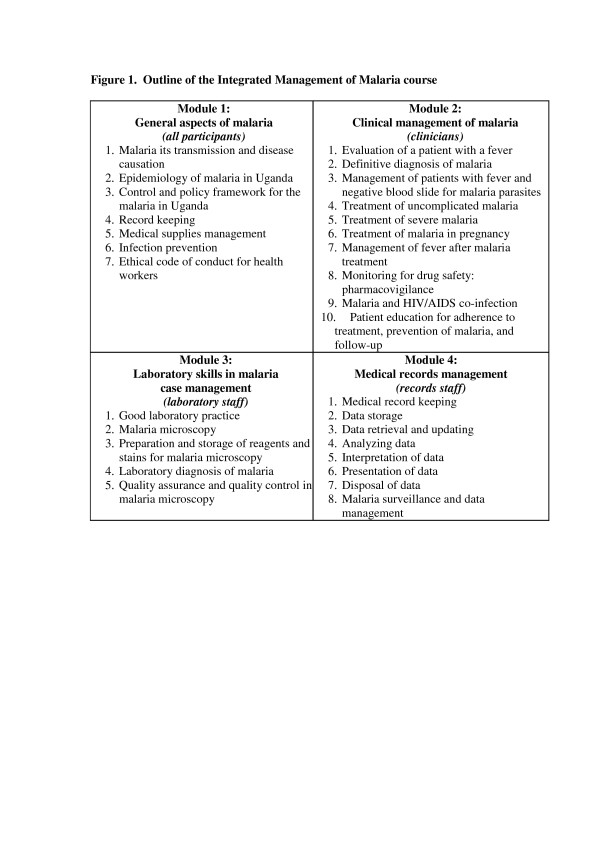
**Outline of the Integrated Management of Malaria course**.

The Integrated Management of Malaria course was designed for teams of clinicians, laboratory professionals, and health information assistants with the goal of improving the management of febrile patients, and encouraging communication and trust among people responsible for different tasks. The curriculum and training materials were developed by JUMP through an interactive process described in Ssekabira et al. [11]. The six-day course included both didactic and practical sessions, as outlined in Figure 1. Clinical placements were designed to allow clinicians to observe management of very sick children. Laboratory placements were designed to allow laboratory professionals to apply skills learnt in classroom sessions. To minimize disruption of patient services, the staff at each site was divided into two groups and trained in two contiguous sessions of the course.

Three support supervision visits were conducted by the JUMP mobile team approximately six weeks, 12 weeks and one year after the course, to provide feedback and reinforce training messages, as well as perform the on-site observation (see below).

### Evaluation

#### Clinical skills assessment

Two clinical observation tools were developed for children less than five years of age and patients five years or more of age. The tool for children less than five years of age was based on the Integrated Management of Childhood Illness tools, with adaptations for laboratory diagnosis of malaria. A similar tool was developed for patients five years or more of age. The tools were pilot tested in Kampala City Council health centres that were not UMSP sites, and revised appropriately. Piloting served the dual purpose of ensuring all relevant information was collected with the tool and enabling the JUMP mobile team to practice using them before going to the sites.

Clinicians were assessed on ability to perform five tasks for patients presenting with fever: proper history taking, thorough physical examination, correct diagnosis, treatment in compliance with national policy, and appropriate patient education. Correct diagnosis was based on the results of microscopy at the health facility and clinical judgment of the JUMP medical officer. Regarding patient education, clinicians were assessed on providing advice to patients and caretakers on malaria treatment and prevention, completion of treatment, identification of warning signs of worsening condition, and use of insecticide-treated bed nets.

The dependent variable for each set of tasks was dichotomous, where one was defined as performing 75% of the tasks correctly for history-taking (asked 12 out of 15 questions), physical examination (performed 15 out of 20 examinations), and patient education (advised on six out of eight topics) and zero otherwise. For diagnosis and treatment, one was simply defined as correct, and zero otherwise.

#### Laboratory skills assessment

Laboratory professionals were assessed on three performance indicators: quality of malaria blood smear prepared, and sensitivity and specificity of malaria blood smear results. Each laboratory professional prepared five thick smears stained with 10% Giemsa and five thin smears under observation by the JUMP laboratory technologist. Smears were assessed on eight criteria, four of which were common to thick and thin smears: 1) size (diameter of approximately 10 mm); 2) minimum or no artifacts; 3) uniform thickness; 4) quality of the stained blood smears (white blood cell nuclei deeply stained, a purple background, with visible eosinophilic granules, parasite cytoplasm and chromatin). The four additional criteria for thick smears were: spread neither 1) too thick nor 2) too thin as judged by the legibility of the printed text seen through the slide; 3) complete lysis of red blood cells; and 4) blue background, blue neutrophil nuclei and orange granules, blue eosinophil nuclei and reddish orange granules. The four additional criteria for thin smear were: 1) no feathered edge; 2) red blood cells not distorted; 3) absence of severe clumping; and 4) red blood cells not distributed into a single layer.

Laboratory professionals were also asked to interpret 30 quality control slides for malaria blood smears: 15 positive and 15 negative. The 15 positive smears represented a range of low, medium and high parasite densities and examples of less prevalent species in addition to *P. falciparum*. Their smear results were cross-checked by the JUMP laboratory technologist to ascertain sensitivity and specificity.

#### On-site observation schedule

JUMP's three-person mobile team was comprised of a medical officer, laboratory technologist and data management specialist. Before the six-day course, the mobile team performed baseline on-site observation of clinical care and laboratory testing, which also served as a learning-needs assessment. In addition, trainees were recruited and briefed about the overall training programme. At baseline, departments within the sites operated independently at each step of patient management. For example, records were maintained by each department individually that were inconsistent, repetitive and showed little communication among departments. At some sites, clinicians wrote requests for laboratory investigations simultaneously with prescriptions.

After the six-day course, the JUMP mobile team made three follow-up visits approximately six weeks (three to nine weeks), 12 weeks (10 to 17 weeks) and one year (11 to 23 months) later. Table S1 of the Additional file 1 presents dates of the six-day course, and baseline and follow-up visits. They performed on-site observation of clinical care and laboratory testing, and then provided individual feedback and reinforced training messages. Observations were performed before support activities, so trainees were not "primed" by a mentoring session immediately before on-site observation.

#### Data management and statistical analysis

Data were entered into EPIINFO 6.0 and analyzed using Stata version 11.0 (StataCorp LP, College Station TX, 2009), and Microsoft Office Excel 2007, (Microsoft Corporation, Redmond WA, 2007). For clinical skills assessment, unadjusted relative risks were calculated using the cohort study (CS) command, comparing baseline to six week, 12-week and one-year follow-up, i.e. ratio of the percentage performed correctly at follow-up to the percentage at baseline. The same command was used to compare six week to 12-week follow-up and 12-week to one-year follow-up. For laboratory skills observation, comparisons were based on a chi-square test.

Adjusted comparisons that controlled for unobserved individual effects were performed with the XTGEE command; this accounted for clustering when one clinician was observed at more than one visit as well as seeing more than one patient during each visit. The estimates were performed with binomial family, link = log. The correlation matrix was exchange, because the sample was unbalanced, i.e. the number of clinician-patient encounters observed was not the same for each clinician nor each point in time. Estimated coefficients were exponentiated to report the relative risk with the eform option, which displays exponentiated coefficients). All tests for significance were based on an alpha of 0.05.

#### Approval

The final Integrated Management of Malaria curriculum, training materials and evaluation tools were approved by the NMCP, Uganda's National Malaria Case Management Technical Working Group, and other stakeholders. The curriculum was also adopted by the STOP Malaria Project and additional training was funded by the US President's Malaria Initiative.

## Results

### Descriptive statistics

From December 2006 to June 2007, 194 health professionals participated in the Integrated Management of Malaria course at the IDI in Kampala, of whom 118 were clinicians, 30 were laboratory staff, 32 were records staff, and 14 were district health officers.

Table 1 describes malaria endemicity of the site, profession, and gender of the 118 clinicians who were trained, 101 clinicians who were observed, and two subsamples: 1) the panel of 61 clinicians who were observed at baseline and at least once after the training, and 2) the clinicians who were observed but did not meet the criteria for the panel. The distribution of clinicians by malaria endemicity, profession and gender was not statistically significantly different between the trainees and sample of clinicians who were observed. The results were similar for the comparison of clinicians in the panel and those not in the panel, with the exception that the percentage of males was statistically significantly higher in the panel than the other subsample. Table S1 of the Additional file 1 reports the number of clinicians observed at each site.

**Table 1:** Characteristics of clinicians who were trained, observed and in panel with matched observations

	Trained	Observed	Panel*	Not panel*
Sample Size	118	101	61	40
Endemicity at site [17,18]				
EIR less than 1	24 (20)	17 (17)	12 (20)	5 (13)
EIR 3-7	58 (49)	48 (48)	25 (41)	23 (58)
EIR greater than 365	36 (30)	36 (36)	24 (39)	12 (30)
Profession				
Doctors	10 (8)	6 (6)	4 (7)	2 (5)
Clinical officers	27 (23)	27 (27)	21 (34)	6 (15)
Nurses/midwives	79 (67)	68 (67)	36 (59)	32 (80)
Other clinical staff	2 (2)	0 (0)	0 (0)	0 (0)
Gender				
Male	49 (42)	42 (42)	31 (51)	11 (28)
Female	70 (58)	59 (58)	30 (49)	29 (73)

*Panel denotes individuals who had visit data from baseline and at least one follow-up at six weeks, 12 weeks or one year

EIR means Entomological Inoculation Rate per year

More information on the EIR, dates of observation, and panel of clinicians observed at each of the sites is presented in Table S1 of the Additional file 1

The laboratory professionals were technologists, technicians and assistants. For laboratory professionals, 17 out of 30 people trained (57%) were observed.

### Clinical skills

**Figure 2:**
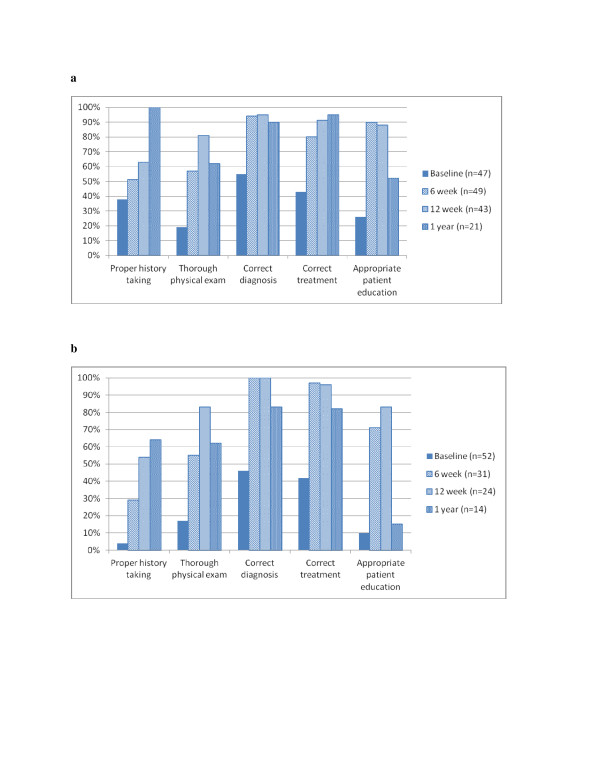
**a Effect of Integrated Management of Malaria course on performance of five key tasks for patient less than five years of age presenting with fever b Effect of Integrated Management of Malaria course on performance of five key tasks for patients five years or more of age presenting with fever**.

Performance of five key skills for patients presenting with fever improved between baseline and each of three follow-up visits. Figure 2a and 2b report the percentage of tasks performed correctly by age group. In analyses of unadjusted relative risk, all increases were statistically significant with two exceptions: 1) history-taking for children less than five years of age from baseline to six week follow-up (38% v 51%, RR = 1.33, CI 0.84,2.10) and patient education for patients five years or more of age from baseline to one year follow-up (10% v 15%, RR = 1.60, CI 0.35,7.34).

Two estimates of relative risk are compared in Table 2: 1) unadjusted model and unmatched sample similar to those in Figure 2a and 2b, and 2) model that adjusted for unobserved differences across clinicians and matched sample. In Table 2, the matched sample means that estimates were based on a sub-sample of clinicians who were observed at baseline and the relevant follow-up visit. The estimates combined observations across age groups at each visit. All increases were statistically significant with one exception; the adjusted model for patient education from baseline to one year follow-up (17% v 38%, RR = 2.04, CI 0.97,4.26). These results and their confidence intervals are reported in Table S2 of the Additional file 2.

**Table 2:** Comparison of relative risk estimates in unadjusted model with unmatched sample to adjusted model with matched sample at each time point

Indicator	Percentage correct at six weeks relative to baseline			Percentage correct at 12 weeks relative to baseline			Percentage correct at one year relative to baseline		
	**Unadjusted**	**Adjusted**	**Difference**	**Unadjusted**	**Adjusted**	**Difference**	**Unadjusted**	**Adjusted**	**Difference**
					
	**RR**	**RR**		**RR**	**RR**		**RR**	**RR**	

Number of patients observed	179	124		166	109		133	56	

Proper history taking	2.10	1.86	-0.24	2.96	3.35	0.39	4.37	5.63	1.26

Thorough physical exam	3.09	2.66	-0.43	4.51	4.09	-0.42	3.40	2.35	-1.05

Correct diagnosis	1.91	1.77	-.14	1.92	1.96	0.04	1.74	2.16	0.42

Correct treatment	2.03	1.96	-.07	2.18	2.42	0.24	2.14	2.21	0.07

Appropriate patient education	4.80	4.47	-.33	5.04	5.74	0.70	2.23	2.04	-0.19

The unadjusted model was estimated with unmatched observations. The adjusted model was estimated with matched observations

These results and their confidence intervals are reported in Table S2 of the Additional file 2. All increases were statistically significant with the exception of patient education from baseline to one year when estimated with the adjusted model

The number of clinician as well as the number of patients observed was smaller in the matched than the unmatched sample. For the percentage correct at six weeks relative to baseline, 49 clinicians were observed in the matched compared to 94 in the unmatched sample. For the percentage correct at twelve weeks relative to baseline, 47 clinicians were observed in the matched compared to 95 in the unmatched sample. For the percentage correct at one year relative to baseline, 24 clinicians were observed in the matched compared to 84 in the unmatched sample

Results of the comparison of estimates from the unadjusted model and unmatched sample to the adjusted model and matched sample were heterogeneous, as shown in Table 2. For the improvement from baseline to six weeks, the unadjusted model and unmatched sample overestimated the effects of training for all five tasks. In contrast, for the improvement from baseline to 12 weeks, the unadjusted model and unmatched sample underestimated the effects of training for four out of five tasks. For the improvement from baseline to one year, it under-estimated the effects of training for three out of five tasks. The increase in the percentage of patients that had a thorough physical examination was over-estimated by the unadjusted model and unmatched sample at all three points in time.

Tests were conducted for whether or not the improvement at six-week follow-up were maintained at 12 weeks and one year. In estimates with the unadjusted model and unmatched sample, the percentage of patients with proper history-taking continued to increase significantly from six to 12 weeks and 12 weeks to one year (43% v 60%, RR = 1.40, CI 1.02,1.94; 60% v 89%, RR = 1.48, CI 1.17,1.86). Thorough physical examination continued to increase significantly from six to 12 weeks (56% v 82%, RR = 1.46, CI 1.17,1.82), but then decreased significantly from 12 weeks to one year (82% v 60%, RR = 0.75, CI 0.56,1.00). Improvements in correct diagnosis and correct treatment at six weeks were maintained from six to 12 weeks and 12 weeks to one year (diagnosis: 96% v 97% v 88%, RR = 1.01 CI 0.95,1.07, RR = 0.91 CI 0.79,1.03; treatment: 86% v 93% v 91%, RR = 1.07 CI 0.96,1.20, RR = .97 CI 0.86,1.12). Improvements in the percentage of patients with appropriate patient education were maintained from six to 12 weeks (83% v 87%, RR = 1.04, CI 0.91,1.20), but then decreased significantly from 12 weeks to one year (87% v 38%, RR = 0.44, CI 0.29,0.68).

Results for Kasambya were not reported Ssekabira et al. [11]. For the sake of comparison to the surveillance data, the analyses were repeated with Kasambya excluded and results were similar those presented above.

### Laboratory skills

Preparation of malaria blood smears improved significantly from baseline to six weeks, 12 weeks, and one year, as shown in Table 3. Note that one year follow-up data were for thick smears only. The sensitivity of interpreting smear results increased from baseline to each follow-up visit, and the difference was statistically significant at one year (48.6% to 84.1%, *p *< 0.036). Specificity also increased from baseline to each follow-up visit, but none of the increases were statistically significant.

**Table 3:** Percentage of laboratory tasks were performed correctly

	Percentage of tasks correct				Relative Risk (unadjusted and unmatched sample)		
**Indicator**	**Baseline**	**Six week**	**12 week**	**One year**	**Percentage correct at six weeks relative to baseline**	**Percentage correct at 12 weeks relative to baseline**	**Percentage correct at one year relative to baseline**

Smear preparation	21.6	67.3**	63.2*	60.0*	3.12	2.93	2.78

Sensitivity	48.6	70.6	69.9	84.1*	1.45	1.44	1.73

Specificity	72.1	77.2	90.6	94.2	1.07	1.26	1.31

P-values are for comparison between performances at baseline and follow up in health facility:*p < 0.05; **p < 0.01

The number of laboratory professionals observed was 17 at baseline, 16 at six weeks, 14 at 12 weeks, and 15 at one year

## Discussion

JUMP was associated with statistically significant progress towards treatment for malaria according to NMCP recommendations at UMSP sites. Significant improvements relative to baseline were observed for all five tasks for patients presenting with fever during almost every follow-up visit, i.e. 14 out of 15 comparisons of the matched sample and adjusted estimates. Among laboratory professionals, significant improvements relative to baseline were observed in malaria blood smear preparation at every follow-up visit, and in sensitivity of interpretation of test results at one-year follow-up.

Considering the role of individual effects in a pre/post evaluation design, results from the estimates that were adjusted for individual effects in the matched sample suggested that differences in practice style across clinicians may bias the evaluation of training programmes, especially with regard to performing a thorough physical examination. Data were not available for a similar analysis of laboratory professionals and would be worth pursuing in the future.

These clinical results were supported by UMSP surveillance data on clinic-level performance indicators during the four months after the initial JUMP training when the six and 12-week follow-up visits occurred [11], and 11 to 23 months after the initial JUMP training when the third follow-up visit occurred [12]. See Table three [12] for Kamwezi and Kihihi Oct-Dec 06 to Oct-Dec 08, Aduku Jan-Mar 07 to Jan Mar 08, and Nagongera and Walakuba Apr-Jun 07 to Oct-Dec 08.

UMSP continued monthly or bi-monthly site visits at six of the nine sites through at least March 2010, which could also be considered an intervention. It was not possible to distinguish the effects of JUMP from UMSP, because they were implemented jointly at the same sites. The Integrated Infectious Disease Capacity-Building Evaluation (IDCAP) is a randomized controlled trial that seeks to distinguish their effects. A health management information system based on the UMSP patient record form was introduced at 36 sites in Uganda. Eighteen sites were randomly assigned to receive on-site support for nine months, which included team training, clinical coaching, and continuous quality improvement. Results will be available later this year.

JUMP's laboratory skills assessment showed large improvements in the sensitivity (absolute increases of 21 to 35%) and specificity (five to 23%) of interpreting malaria blood smear results that were statistically significant in only one out of six comparisons. The laboratory skills assessment results were not strictly comparable to the quality assurance data reported in Ssekabira et al. [11] which showed the sensitivity and specificity of field microscopy did not improve significantly with training, and Kiggundu et al. [7] which showed they did. In Ssekabira et al. [11] and Kiggundu et al. [7], blood slides were collected before and after training which reflected the parasite densities of the clinic patient population, and then sent to a central laboratory to be read by an expert. Baseline sensitivity was 86% and specificity was 90% in Ssekabira et al. [11], and 84% and 87%, respectively, in Kiggundu et al. [7]. Note that these levels of accuracy were the basis for diagnosing and treating patients during the clinical skills assessment. For the laboratory skills assessment, JUMP trainees read quality control slides that included a range of parasite densities and less prevalent species of malaria, which appear in fewer than 5% of cases. Baseline sensitivity was 48% and specificity was 72%.

With the JUMP laboratory skill assessment, sensitivity reached 84% at one year and specificity reached 91% after 12 weeks, which was maintained at one year. WHO recommended that laboratory personnel should be able to detect the presence or absence of malaria parasites with 90% accuracy [22]; that standard was met for specificity, but not sensitivity. The example of quality control slides that WHO cited for accrediting laboratory personnel was similar to the JUMP slides [23].

## Limitations

The main limitation of the evaluation was that health professionals were aware they were under observation and may have modified their usual behaviour. Leonard and Masatu [24] reported a significant improvement in quality during observation of clinical care by comparing patient reports on visits before the observation with their reports during observation. They also reported that quality during observation decreased with the number of patients observed. In this evaluation, the Hawthorne effect would have biased the on-site observation at baseline as well as follow-up, so that the results may have accurately measured the improvements in skills if not practice. The surveillance data may have served as a measure of the effect on actual practice.

In addition, the intervention did not address all health system factors that usually affect quality of care such as supply chain management, and unreliable power supply in the laboratory. To achieve maximum benefits, capacity-building and surveillance interventions must be accompanied by strategies to address underlying health system issues.

Finally, the Integrated Management of Malaria course was six days, which may appear expensive to bring to scale. Given evidence that shorter courses or courses for individual clinicians were not effective [3,6], a longer, team-based training program could potentially be cost-effective.

## Conclusion

The Integrated Management of Malaria course was associated with statistically significant improvements in case management of patients presenting with fever, as measured by both on-site clinical observation and surveillance data. These improvements persisted for at least one year after the six-day course. The results of on-site laboratory observation also suggested improved performance of laboratory staff in malaria microscopy in contrast to the laboratory quality assurance data. As a joint programme, the effects of the course can not be distinguished from the UMSP activities, but lend support to long-term, on-going capacity-building and surveillance interventions.

## Abbreviations

ACT: Artemisinin-based Combination Therapy; AL: Artemether and Lumefantrine; EIR: Entomological Inoculation Rate; IDI: Infectious Diseases Institute; JUMP: Joint Uganda Malaria Training Program; NMCP: National Malaria Control Program; UMSP: Uganda Malaria Surveillance Program; WHO: World Health Organization.

## Competing interests

Drs Gisela Schneider and Lydia Mpanga Sebuyira state that under their successive leadership, the IDI training programme received funds from various pharmaceutical companies and public agencies sources, none of which they believe present any conflicts of interest. Kelly Willis, Senior Vice President at Accordia Global Health Foundation states that a grant from ExxonMobil was received for development and implementation of the Integrated Management of Malaria course, which she does not believe was a conflict of interest for her staff.

## Authors' contributions

Conception and design: AN, AO, GS, US, MRW. Development of Integrated Management of Malaria course: GD, LMS, AO, GS, US, AY. Data collection: AN, AO, US, AY. Data analysis and interpretation: SB, AN, MRW. Drafting of paper: NB, SB, GD, AN, AO, US, MRW, AY. Revision and approval of final draft: All authors

## Supplementary Material

Additional file 1 **Table S1**. Characteristics of sites including EIR, dates of training and visits, and clinicians in the panel. Click here for file

Additional file 2 **Table S2**. Comparison of relative risk estimates (with confidence intervals) in unadjusted model with unmatched sample to adjusted model with matched sample at each time point. Click here for file
