# Supplementary material for: Improved clinical and laboratory skills after team-based, malaria case management training of health care professionals in Uganda
Source: Malar J. 2012 Feb 13;11:44. doi: 10.1186/1475-2875-11-44 (PMC3342908; doi:10.1186/1475-2875-11-44)
Supplement: Additional file 1 — Table S1. Characteristics of sites including EIR, dates of training and visits, and clinicians in the panel. [file 1475-2875-11-44-S1.DOC]

**Table S1. Characteristics of sites including entomologic inoculation rate, dates of training and visits, and clinicians in the panel**

|  | **Kabale** | **Kamwezi** | **Kasambya** | **Kihihi** | **Walukuba** | **Kyenjojo** | **Omugo** | **Nagongera** | **Aduku** |
| --- | --- | --- | --- | --- | --- | --- | --- | --- | --- |
| EIR* [17, 18] | <1 | <1 | 3 | 6 | 6 | 7 | 397 | 562 | 1586 |
| Baseline visit | 21-Sep-06 | 22-Sep-06 | 23-Feb-07 | 18-Sep-06 | 14-May-07 | 19-Feb-07 | 12-Jan-07 | 10-May-07 | 8-Sep-06 |
| Last date of training | 20-Dec-06 | 20-Dec-06 | 31-Mar-07 | 20-Dec-06 | 9-Jun-07 | 31-Mar-07 | 14-Feb-07 | 9-Jun-07 | 14-Feb-07 |
| 1^st^ follow-up | 19-Jan-07 | 22-Jan-07 | 30-May-07 | 18-Jan-07 | 23-Jul-07 | 31-May-07 | 19-Mar-07 | 25-Jul-07 | 6-Mar-07 |
| 2^nd^ follow-up | 16-Apr-07 | 17-Apr-07 | 17-Jul-07 | 12-Apr-07 | 4-Sep-07 | 9-Jul-07 | 30-Apr-07 | 7-Sep-07 | 26-Apr-07 |
| 3^rd^ follow-up | 23-Oct-08 | 27-Oct-08 | None | 20-Oct-08 | 30-Oct-08 | None | 5-Jan-08 | 13-Nov-08 | 8-Jan-08 |
| **Profession** |  |  |  |  |  |  |  |  |  |
| Doctors | 2 | 0 | 0 | 0 | 1 | 0 | 0 | 0 | 1 |
| Clinical officers | 5 | 1 | 1 | 1 | 2 | 3 | 2 | 4 | 2 |
| Nurses and midwives | 0 | 3 | 4 | 6 | 4 | 4 | 6 | 8 | 1 |
| **Gender** |  |  |  |  |  |  |  |  |  |
| Male | 7 | 2 | 1 | 2 | 3 | 2 | 5 | 6 | 3 |
| Female | 1 | 2 | 4 | 5 | 3 | 5 | 3 | 6 | 1 |
| **Overall** | **8** | **4** | **5** | **7** | **6** | **7** | **8** | **12** | **4** |

**Legend for Table S1**

EIR means Entomological Inoculation Rate

able 2ed.Uganda excluded

and observation aseline and at least ) don'e. they weren'sit were maintained through the one year fol….
