# Supplementary material for: Improved clinical and laboratory skills after team-based, malaria case management training of health care professionals in Uganda
Source: Malar J. 2012 Feb 13;11:44. doi: 10.1186/1475-2875-11-44 (PMC3342908; doi:10.1186/1475-2875-11-44)
Supplement: Additional file 2 — Table S2. Comparison of relative risk estimates (with confidence intervals) in unadjusted model with unmatched sample to adjusted model with matched sample at each time point. [file 1475-2875-11-44-S2.DOC]

**Table S2. Comparison of relative risk estimates (with confidence intervals)** in unadjusted model with unmatched sample to adjusted model with matched sample at each time point

|  | Percentage correct at 6 weeks relative to baseline | | | | | Percentage correct at 12 weeks relative to baseline | | | | | Percentage correct at 1 year relative to baseline | | | | |
| --- | --- | --- | --- | --- | --- | --- | --- | --- | --- | --- | --- | --- | --- | --- | --- |
|  | Unadjusted, unmatched sample  n=179 | | Adjusted, matched sample  n=124 | | Diff-er-ence | Unadjusted, unmatched sample  n=166 | | Adjusted, matched sample  n=109 | | Diff-er-ence | Unadjusted,unmatched sample  n=133 | | Adjusted, matched sample  n=56 | | Diff-er-ence |
| Key task | RR | CI | RR | CI |  | RR | CI | RR | CI |  | RR | CI | RR | CI |  |
| Proper history taking | 2.10 | 1.32, 3.36 | 1.86 | 1.20, 2.88 | -0.24 | 2.96 | 1.91, 4.58 | 3.35 | 1.95, 5.74 | 0.39 | 4.37 | 2.90, 6.58 | 5.63 | 2.53, 12.54 | 1.26 |
| Thorough physical exam | 3.09 | 1.95, 4.90 | 2.66 | 1.60, 4.41 | -0.43 | 4.51 | 2.93, 6.96 | 4.09 | 2.47, 6.78 | -0.42 | 3.40 | 2.07, 5.57 | 2.35 | 1.38, 3.98 | -1.05 |
| Correct diagnosis | 1.91 | 1.56, 2.33 | 1.77 | 1.41, 2.23 | -.14 | 1.92 | 1.57, 2.34 | 1.96 | 1.53, 2.52 | 0.04 | 1.74 | 1.38, 2.20 | 2.16 | 1.38, 3.37 | 0.42 |
| Correct treatment | 2.03 | 1.59, 2.60 | 1.96 | 1.46, 2.63 | -.07 | 2.18 | 1.72, 2.77 | 2.42 | 1.75, 3.34 | 0.24 | 2.14 | 1.66, 2.76 | 2.21 | 1.44, 3.38 | 0.07 |
| Appropriate patient education | 4.80 | 3.08, 7.49 | 4.47 | 2.68, 7.46 | -.33 | 5.04 | 3.24, 7.85 | 5.74 | 3.13, 10.52 | 0.70 | 2.23 | 1.21, 4.09 | 2.04 | .97, 4.26 | -0.19 |

**Legend for Table S2**

The number of clinician as well as the number of patients observed was smaller in the matched than the unmatched sample. For the percentage correct at six weeks relative to baseline, 49 clinicians were observed in the matched compared to 94 in the unmatched sample. For the percentage correct at twelve weeks relative to baseline, 47 clinicians were observed in the matched compared to 95 in the unmatched sample. For the percentage correct at one year relative to baseline, 24 clinicians were observed in the matched compared to 84 in the unmatched sample.
